# Tumor vasculature remolding by thalidomide increases delivery and efficacy of cisplatin

**DOI:** 10.1186/s13046-019-1366-x

**Published:** 2019-10-28

**Authors:** Yanwei Shen, Shuting Li, Xin Wang, Mengying Wang, Qi Tian, Jiao Yang, Jichang Wang, Biyuan Wang, Peijun Liu, Jin Yang

**Affiliations:** 1 Department of Medical Oncology, First Affiliated Hospital of Xi’an Jiaotong University, No. 277 of the Western Yanta Road, Xi’an, 710061 Shaanxi China; 2 Key Laboratory of Environment and Genes Related to Diseases, Xi’an Jiaotong University Health Science Center, Xi’an, 710061 Shaanxi China; 3 Department of Vascular Surgery, First Affiliated Hospital of Xi’an Jiaotong University, Xi’an, 710061 Shaanxi China; 4 Center for Translational Medicine, First Affiliated Hospital of Xi’an Jiaotong University, No. 277 of the Western Yanta Road, Xi’an, 710061 Shaanxi China; 5 Key Laboratory for Tumor Precision Medicine of Shaanxi Province, First Affiliated Hospital of Xi’an Jiaotong University, No. 277 of the Western Yanta Road, Xi’an, 710061 Shaanxi China

**Keywords:** Thalidomide, Vascular normalization, Solid tumor, Drug delivery, Chemoresistance

## Abstract

**Background:**

A promising strategy to overcome the chemoresistance is the tumor blood vessel normalization, which restores the physiological perfusion and oxygenation of tumor vasculature. Thalidomide (Thal) has been shown to increase the anti-tumor effect of chemotherapy agents in solid tumors. However, it is not yet known whether the synergistic effect of Thal combined with other cytotoxic drugs is attributable to tumor vascular normalization.

**Methods:**

We used two homograft mice models (4 T1 breast tumor model and CT26 colorectal tumor model) to investigate the effect of Thal on tumor growth, microvessel density, vascular physiology, vascular maturity and function, drug delivery and chemosensitivity. Immunofluorescence, immunohistochemistry and scanning electron microscopy were performed to determine the vessel changes. Protein array assay, qPCR and western blotting were used to detect the molecular mechanism by which Thal regulates tumor vascular.

**Results:**

Here we report that Thal potently suppressed tumor growth, angiogenesis, hypoxia, and vascular permeability in animal models. Thal also induced a regular monolayer of endothelial cells in tumor vessels, inhibiting vascular instability, and normalized tumor vessels by increasing vascular maturity, pericyte coverage and endothelial junctions. The tumor vessel stabilization effect of Thal resulted in a decrease in tumor vessel tortuosity and leakage, and increased vessel thickness and tumor perfusion. Eventually, the delivery of cisplatin was highly enhanced through the normalized tumor vasculature, thus resulting in profound anti-tumor and anti-metastatic effects. Mechanistically, the effects of Thal on tumor vessels were caused in part by its capability to correct the imbalance between pro-angiogenic factors and anti-angiogenic factors.

**Conclusions:**

Our findings provide direct evidence that Thal remodels the abnormal tumor vessel system into a normalized vasculature. Our results may lay solid foundation for the development of Thal as a novel candidate agent to maximize the therapeutic efficacy of chemotherapeutic drugs for solid tumors.

**Electronic supplementary material:**

The online version of this article (10.1186/s13046-019-1366-x) contains supplementary material, which is available to authorized users.

## Background

Tumor angiogenesis, the formation of new vascular network, is a well-recognized hallmark of cancer [1]. Growth, proliferation and metastasis of tumor strongly depends on the expansion of the host vasculature, which not only supplies nutrients required for tumor progression and drains away wastes produced by cancer cells, but also provides cancer cells with a metastatic route for colonizing distant organs [2, 3]. Hence, interrupting this process has been considered a promising therapeutic strategy for the treatment of tumors. Traditional anti-angiogenic therapies have focused mainly on the suppression of new vessel formation and destruction of pre-existing tumor vessels to starve and deprive the tumor from its nutrient supply [4–6]. Because of its central role in pathological angiogenesis, vascular endothelial growth factor (VEGF) and its receptor VEGFR2 have been key targets of anti-angiogenic drug development [7]. Recent evidences have demonstrated that inhibition of angiogenesis suppresses tumor growth and metastasis [8, 9], whereas others have shown that anti-angiogenic approaches enhanced intratumoral hypoxia resulting from vascular regression and rarefaction, and eventually lead to increased resistance to treatment, local tumor invasion into surrounding tissue, and frequency of metastasis [9, 10]. Transient benefits of anti-angiogenic agents are rapidly followed by tumor relapse, sometimes associated with establishment of resistance and heightened tumor growth and malignancy [11, 12]. Therefore, increasing effort has been made to better understand the biological processes that underlie tumor vascularization and lay the foundation for additional angiogenesis targeted therapies.

Indeed, tumor vessels exhibit altered behaviors and are functionally distinct from the normal vasculature. During tumor progression, it has been described an alteration of the balance of pro-angiogenic and anti-angiogenic signaling and a relentless production of angiogenic stimulators in the tumor context. This unbalance induces the formation of immature, malformed, disorganized and tortuous tumor vessels, lacking tight association between pericytes and endothelial cells layer and unable to efficiently deliver oxygen [13, 14]. The dysfunctionality of tumor vasculature has profound consequences for the tumor microenvironment (TME) and convert the tumor into a hostile hypoxic and acidic milieu surrounded by high interstitial fluid pressure (IFP), which acts as a pathologic barrier to drug delivery into tumors [15, 16].

Given the role of abnormal tumor vascular system in regulating adverse TME, in the last decades, there have been many groups trying to “normalize” the tumor microvessels so as to improve the TME [16]. Emerging preclinical and initial clinical evidence has shown that normalization of disorganized tumor vasculature using therapeutics, rather than the blockage or disruption of tumor blood vessels, reduced number and size of immature vessels, increased vessel pericyte coverage, and reduced IFP [17–19]. In addition to improving the overall TME, those normalized vessels also make the surviving cells more vulnerable to the treatments that they can deliver more efficiently [20]. Tumor vascular normalization has recently emerged as a complementary therapeutic paradigm to antitumor neovascularization and anticancer therapy.

Thalidomide (Thal), an old drug with a history of nearly sixty years, initially introduced to the market in the 1950s as a sedative for treating nausea in pregnancy, was removed from the market for causing severe congenital limb deformities [21, 22]. Despite its teratogenic properties, Thal has enjoyed a renaissance in recent years. In 1965, an accidental discovery of its immunomodulatory effects was made in patients with erythema nodosum leprosum, thus defining a new indication of usage for Thal [23, 24]. In addition, Thal was found to inhibited induction of the formation of new blood vessels by the basic fibroblast growth factor (bFGF) [25]. In 2006, the use of Thal in combination with dexamethasone has been approved by the Food and Drug Administration (FDA) for the treatment of multiple myeloma [26]. The recognition of Thal as a therapeutic agent provided the impetus for launch of additional studies concerning efficacy of Thal for treating other cancer types, such as prostate cancer, glioblastoma, glioma, renal cell carcinoma, colon cancer and advanced breast cancer [27, 28]. Recently, the Thal has been shown to increase the anti-tumor effect of carboplatin in breast cancer models, but the mechanisms involved in this therapeutic regimen are still poorly elucidated [29]. A better understanding of how Thal enhances therapeutic outcomes of conventional chemotherapeutics is desirable.

This study investigated whether Thal could normalizes tumor vasculatures and generates a favorable TME by enhancing blood perfusion to significantly improve delivery and effcacy of cytotoxic agent. Herein, we uncover that Thal is able to normalize tumor vasculatures for two different mice tumor models including 4 T1 breast tumors and CT26 colorectal tumors, by inhibiting vascular leakage, improving capillary integrity and relieving tumor hypoxia. This was particularly notable for Thal in combination therapy, which increased blood perfusion and led to delayed tumor growth and spreading through improving intratumoral delivery of cytotoxic agent.

## Methods

### Cell lines and reagents

Murine breast cancer 4 T1 and colorectal cancer CT26 cell lines were originally obtained from American Type Culture Collection (ATCC, Manassas, VA, USA), and human umbilical vein endothelial cells (HUVECs) were obtained from Merck Millipore (Temecula, CA, USA). 4 T1 and CT26 cell cultures were maintained using RPMI-1640 medium supplemented with 10% fetal bovine serum (FBS) and 1% penicillin/streptomycin (P/S). HUVECs were cultured in VascuLife VEGF Medium Complete Kit (LL-0003, Lifeline, Frederick, MD, USA). All cells were incubated at 37 °C in humidifed atmosphere of 5% CO_2_. Thal was supplied in gratis by the Changzhou Pharmaceutical Factory (Jiangsu, China). Cisplatin (Cpt) was purchased from Haosoh Pharma (Jiangsu, China). Thalidomide (Thal) was suspended in dimethyl sulfoxide (DMSO), and Cpt was dissolved in distilled saline.

### Cell proliferation and colony formation assay

MTT assay was used to determine the number of viable cells in proliferation. Cells were seeded in 96-well culture plates at a density of 5000 cells/well. After attachment, the culture medium was aspirated and fresh medium with vehicle (0.3% DMSO) or different concentrations of Thal (1, 2, 5 or 10 μg/ml) were added. The MTT solution (Sigma, USA) was added to each well and incubated for 4 h at indicated time points. The absorbance was read at 570 nm with the Multimode Reader (EnSpire PerkinElmer, USA). For colony formation assay, cells (1 × 10^5^/well) were treated with Thal (5 or 10 μg/ml) for 48 h. Then the cells were re-seeded at 1000 cells/well in a 6-well plate and regularly cultured for 14 days. The colonies were stained with crystal violet (Sangon Biotech, China) and counted under a microscope (Olympus BH2, Japan).

### Tumor homograft models

The animal experiments were conducted in accordance with the guidelines of Xi’an Jiaotong University Animal Care and Use Committee. Six to eight weeks old female Balb/c mice weighing 18–20 g in an average were obtained from the Laboratory Animal Center of Xi’an Jiaotong University (Xi’an, China). Animals were acclimatized for 1 week before the start of the experiment. For the 4 T1 breast tumor models, Balb/c mice were inoculated with 1 × 10^6^ 4 T1 cells into the fourth mammary fat pad on the right side. For the CT26 colorectal carcinoma models, 1 × 10^6^ CT26 cells were injected into the right axillary subcutaneous region of mice. The mice bearing subcutaneous CT26 tumor was sacrifced and the tumor mass was resected. Then, the tumor was placed into RPMI-1640 culture medium, and sliced into fragments of 20–30 mm^3^ to make a colon cancer orthograft model. When orthotopic tumor reached approximately a size of 50–100 mm^3^, mice were equally sorted into three groups for 4 T1 (*n* = 6 for each group) and CT26 models (n = 6 for each group), respectively. Treatment groups were intraperitoneally injected with 100 or 200 mg/kg/day of Thal, and control groups were received vehicle (DMSO). Body weight and tumor volume were measured were measured every 2 days. Tumor volumes were calculated based on the formula (a^2^ *×* b)/2 where a is the major tumor axis and b is the minor tumor axis. For combination treatment, 4 T1 tumor-bearing mice were administered vehicle, thalidomide (200 mg/kg/d), CPt (2.5 mg/kg, every other day) or thalidomide + cisplatin intraperitoneally for 28 days. For spontaneous metastasis model, 4 T1 cells were harvested and single-cell suspensions of 1 × 10^6^ cells were directly injected into the fourth mammary fat pat of mice. Mice were euthanized by carbon dioxide chamber at defined time intervals after cell inoculation.

### Histological analyses

All methods for histology and immunostaining have been previously described [30]. Mouse tissue samples were immediately frozen in OCT compound or fixed in 4% PFA overnight at 4 °C, dehydrated and embedded in paraffin. Immunofluorescence was conducted to explore vascular density/areas and vascular normalization. Samples were blocked with 5% goat serum in PBST (0.3% Triton X-100 in PBS) and then incubated for 1 h at room temperature with the following primary antibodies: anti-CD31 (1:75, Abcam, UK), anti-α-SMA (1: 50, Boster, China), anti-claudin-5 (1:100, Abcam, UK), anti-VE-cadherin (1:50, Biolegend, USA) or anti-Ki-67 (1:100, Abcam, UK). After several washes, the samples were incubated for 1 h at room temperature with secondary antibody (diluted 1:200), conjugated to FITC or TRITC (Life Technologies Corporation, USA). Nuclei were counterstained with 4,6-diamidino-2-phenylindole (DAPI). Then the samples were mounted with fluorescent mounting medium and immunofluorescent images were observed under laser confocal microscope (Leica SP5 II, Germany). Tumor necrosis was assessed on H&E stained paraffin sections. Tumor hypoxia was analyzed by immunohistochemistry of HIF-1α (Bioss, China). CPt delivery was quantified by immunostaining of CPt-DNA-adducts (Sigma-Aldrich, Belgium). Tumor vessel perfusion was quantified on tumor cryosections following intravenous injection of 0.05 mg FITC-labeled *Lycopersicon esculentum* lectin (Vector Laboratories, USA) in tumor-bearing mice. Tumor vessel leakage was analyzed on tumor cryosections after intravenous injection of 0.25 mg TRITC-conjugated dextran 40 kDa (Sigma-Aldrich, Belgium) in tumor-bearing mice. Density measurements of blood vessels, necrotic area, pericytes, leakage area, and perfusion area were performed with ImageJ software (http://rsb.info.nih.gov/ij).

### Vessel wall permeability assay

Tumor vessel permeability was detected by an adaptation of the Miles’ assay [31]. Tumor-bearing mice were injected intravenously with 250 μl 0.5% Evans blue (Sigma-Aldrich, Belgium). Thirty minutes later, mice were were sacrificed and tumor lump and liver were resected. The dye was extracted by incubation in 2% formamide at 60 °C and absorbance was read at 610 nm with the Multimode Reader (EnSpire PerkinElmer, USA). The content of Evans blue was expressed by the ratio of tumor to liver content [32].

### Scanning electron microscopy

Tumor-bearing mice were anesthetized and then circulatory perfused by intracardiac injection of 0.1 M phosphate buffer to remove circulating blood. Tumor tissues were broken into small fragments (5 mm × 5 mm) and fixed overnight with 2.5% glutaraldehyde in 0.1 M phosphate buffer (pH 7.2–7.4) at 4 °C. Three rinses of 30 min with 0.1 M phosphate buffer were followed by postfixed with 2% osmiumtetroxide in 0.1 M Na-cacodylate buffer for 2 h at room temperature. Following dehydration in serial dilutionsof hexanenitrile (30, 50, 70 and 100%) the tumor samples were critical-point dried (Balzers, Germany) and mounted on stubs with double-sided adhesive carbon tape. Vascular morphology was observed by scanning electron microscope (JEOL JSM-6360, Japan) at 10 kV.

### RayBiotech mouse chemokine antibody array

Four randomly selected tumor lysates for each group were applied to a Mouse Cytokines Antibody Array (RayBiotech, QAM-CAA-4000, USA) to analyze the level of angiogenesis-related factors according to the manufacturer’s instructions. After development, duplicate films identifying each factor were scanned and images processed and quantified by Genepix 4000B Microarray Scanner.

### Real-time RT-PCR (qPCR)

Total RNA was extracted from the tumor tissues using the TRIzol reagent (Pioneer Biotechnology, China). The cDNA was prepared from total RNA, according to manufacturer’s instructions from the Transcriptor First Strand cDNA Synthesis kit (Roche, Germany). Real-time quantitative PCR was performed using the Real-Time PCR Detection System (Bio-Rad, USA) and SYBR Premix Ex TaqTM II (Takara Bio, USA). After normalizing to the endogenous reference β-actin, the relative expression levels were calculated to represent fold change in gene expression by relative quantification (2^−ΔΔCt^method).

### Western blotting

Western blotting assay was done as previously described [33]. Following primary antibodies were used: anti-ANG1 (1:1000, Abcam, UK), anti-PDGFB (1:500, Cell Signaling Technology, UK) and anti-β-actin (1:5000, Proteintech, China). Secondary antibodies were from Cell Signaling Technology. The reactive bands were visualized by chemiluminescence with the Luminol reagent (Merck Millipore, USA).

### Statistical analysis

Quantitative variables were presented as means ± standard deviations (SD). Statistical significance of difference was evaluated by unpaired Student’s t-tests or one-way ANOVA, as appropriate. The effect of treatment on survival time was determined by log-rank test. All statistical tests were two-sided and the level of significance was set at *p* < 0.05.

## Results

### Thal reduces tumor growth and angiogenesis but improves vascular physiology

**Fig. 1:**
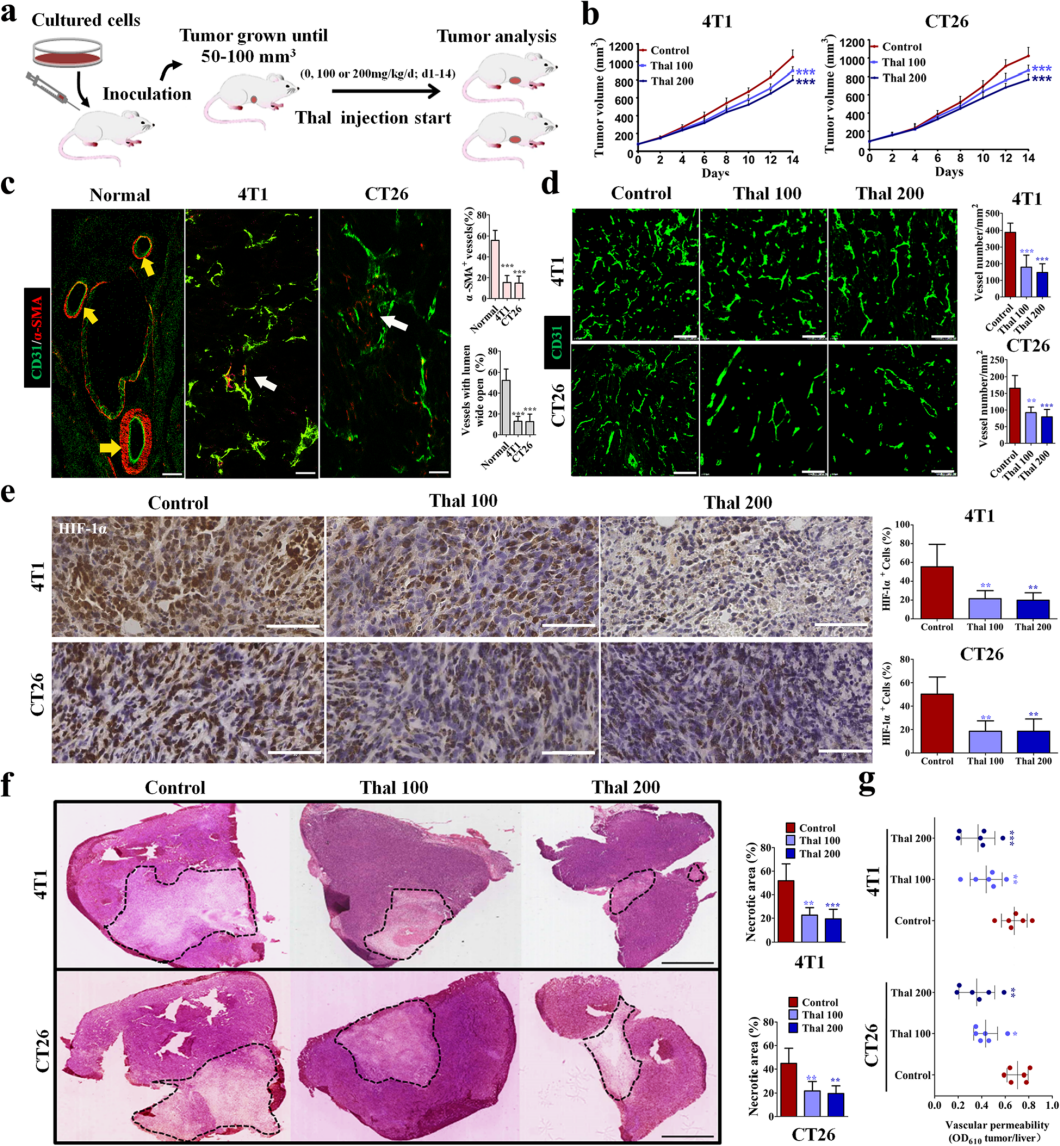
Thal reduces tumor growth, inhibits angiogenesis, and favorably changes the TME. **a** Schematic diagram depicting generation of 4 T1 (*n* = 6) and CT26 (*n* = 6) tumor homograft models and treatment schedule. **b** Tumor growth curves for 4 T1 and CT26 models after treatment with different doses of Thal. **c** Immunostaining for the endothelial cell marker CD31 (Green) and the vessel maturation marker α-SMA (Red) in healthy skin and in tumors (Left), and quantification of percentage of pericyte coverage and proportion of vessels with lumen open (Right). Yellow and white arrows denote mature and immature vessel, respectively. Bars: 50 μm. **d** Confocal micrographs of immunostaining for CD31 (Green) of frozen sections of control and Thal-treated tumors (Left), and quantificationof MVD (Right). Bars: 100 μm. **e** Immunostaining for HIF-1α in tumor tissues from 4 T1 and CT26 models (Left) and quantification of percentage of HIF-1α^+^ cells (Right). Bars: 50 μm. **f** H&E-stained images of tumor sections from 4 T1 and CT26 models and comparison of necrotic areas (black dotted line) between Thal and control; quantification of the necrotic area is indicated as a percentage per total sectional area. Bars: 5 mm. **g** Quantification of vascular permeability in tumors injected with vehicle, Thal 100 mg/kg/day or Thal 200 mg/kg/day. Quantitative data are represented as mean ± SD. All *p* values were calculated by one-way ANOVA analysis followed by Dunnett’s multiple comparison test. *p < 0.05, ** *p* < 0.01, ***p < 0.001

To evaluate the effect of Thal on tumor progression, we implanted 4 T1 cells and CT26 cells in Balb/c mice. When tumors were 50–100 mm^3^, mice were injected with 0, 100, or 200 mg/kg Thal (Fig. 1a). Both 4 T1 and CT26 tumors treated with either 100 mg/kg or 200 mg/kg Thal grew significantly more slowly than control tumors (Fig. 1b). This effect was not consequent to the direct cytotoxicity of Thal, as the Thal did not affect tumor cell proliferation and clonogenicity (Additional file 1: Figure S1). The discrepancy between the effect of thal in vitro and in vivo demonstrate that stromal rather than tumor cell autonomous mechanisms accounted for the anti-tumor effect of Thal in vivo. We thus focused on tumor vasculature because it is known to regulate tumor progression. Double staining for the endothelial cell marker CD31 and mural cell marker α-smooth muscle actin (α-SMA) showed that 4 T1 and CT26 tumor vessels were poorly covered by pericytes, whereas vessels in healthy skin were extensively covered by pericytes, presenting an inerratic lumen morphology (Fig. 1c). These data indicated that vessels in 4 T1 and CT26 orthotopic tumors were immature, thus the two animal models were selected to examine the effect of Thal on tumor vasculature.

We explored the activity of Thal on tumor vascular regression by the staining for microvessels. The staining for microvessels showed that Thal dose-dependently decreased tumor micro-vessel density (MVD) in the 4 T1 model, and similar results were also obtained for CT26 model (Fig. 1d). Meanwhile Thal treatment resulted in decrease of 4 T1 and CT26 tumors hypoxic region (Fig. 1e), as measured by staining of HIF-1α whose accumulation and activity is precisely controlled by the concentration of cellular O_2_, and displayed smaller necrotic areas (Fig. 1f). Moreover, Evans blue penetration assessment indicated that Thal dose-dependently decreased the extravasation of Evans blue dye, implying that the tumor vascular permeability was weaken after Thal treatment (Fig. 1g). Given that a hallmark of various cancers is pathological angiogenesis, which reduces tumor perfusion and converts the tumor into a hostile hypoxic and high interstitial fluid pressure [7], these results are consistent with the conclusion that Thal markedly alters tumor vessel physiology.

### Thal improves tumor vessel maturation

**Fig. 2:**
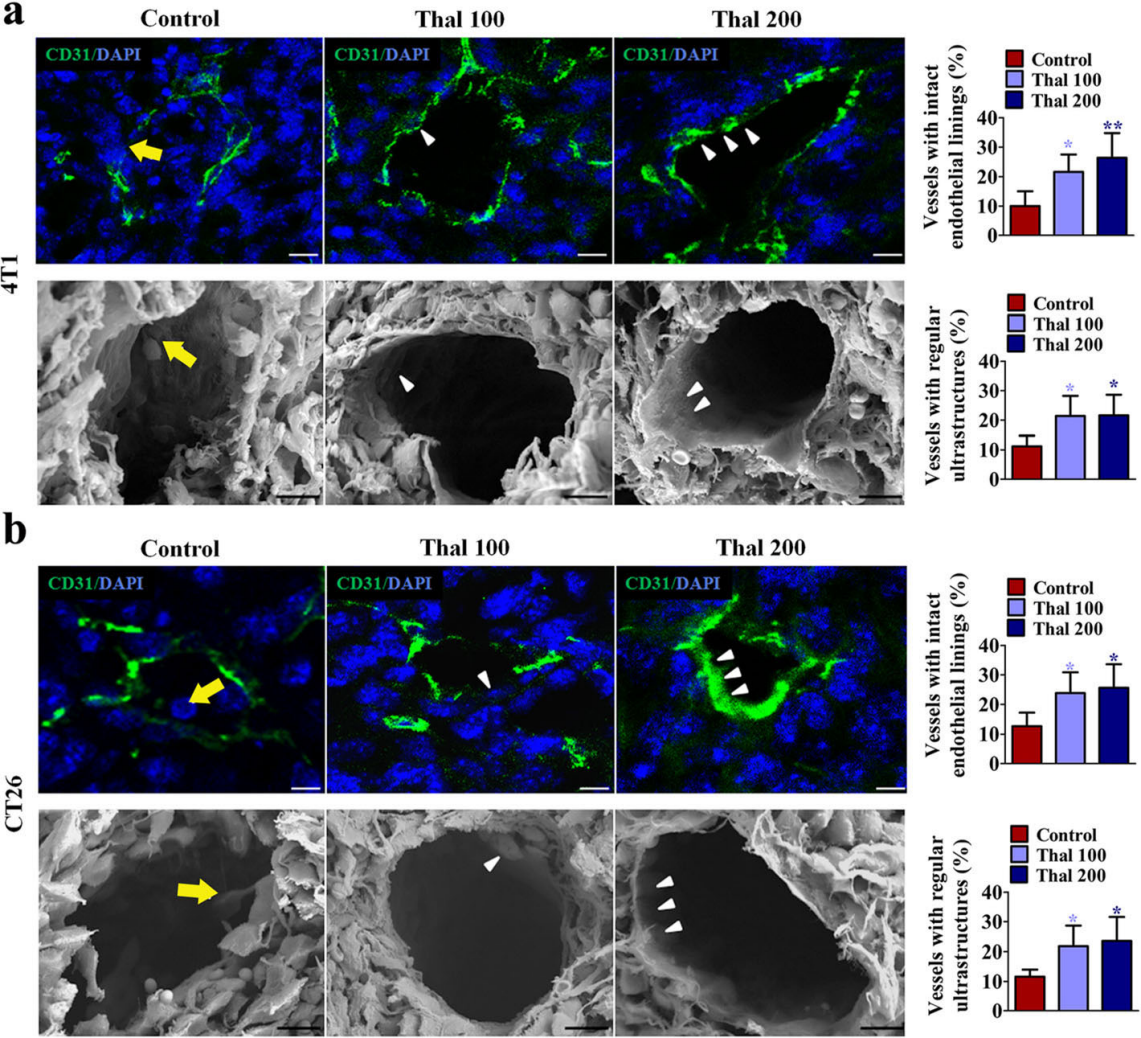
Thal improves the maturity of vascular structure. **a** and **b** Immunostaining of tumor tissues for CD31 and scanning electron microscopy of vessels in control and treated groups from 4 T1 models (**a**) and CT26 models (**b**); quantification of the mature vessels is indicated (percentage of total vessels). Yellow arrows denote chaotically organized endothelial cell linings; white arrowheads indicate smooth thin endothelial linings. Bars: 20 μm (Immunostaining), 10 μm (Scanning electron microscopy). Quantitative data are represented as mean ± SD. All *p* values were calculated by one-way ANOVA analysis followed by Dunnett’s multiple comparison test. *p < 0.05, ** *p* < 0.01

In view of our demonstration that Thal affects tumor vessel physiological characteristics, we further investigated the tumor vessel morphology using CD31 staining and scanning electron microscopy (Fig. 2). Staining for CD31 showed that 4 T1 tumors exhibited thick, irregular vessel walls with endothelial cells extension protruding into the lumen, whereas Thal-treated vessels displayed more uniform alignment of endothelial cells; scanning electron microscopy showed a regular, flat monolayer of endothelial cells in vessels of Thal-treated 4 T1 tumors, in contrast to the more irregular and chaotically organized endothelial cell linings in vessels of control (Fig. 2a). In parallel, similar results were also noted in CT26 tumors (Fig. 2b), implying that Thal possesses the ability to induce vessel normalization on endothelial cells.

**Fig. 3:**
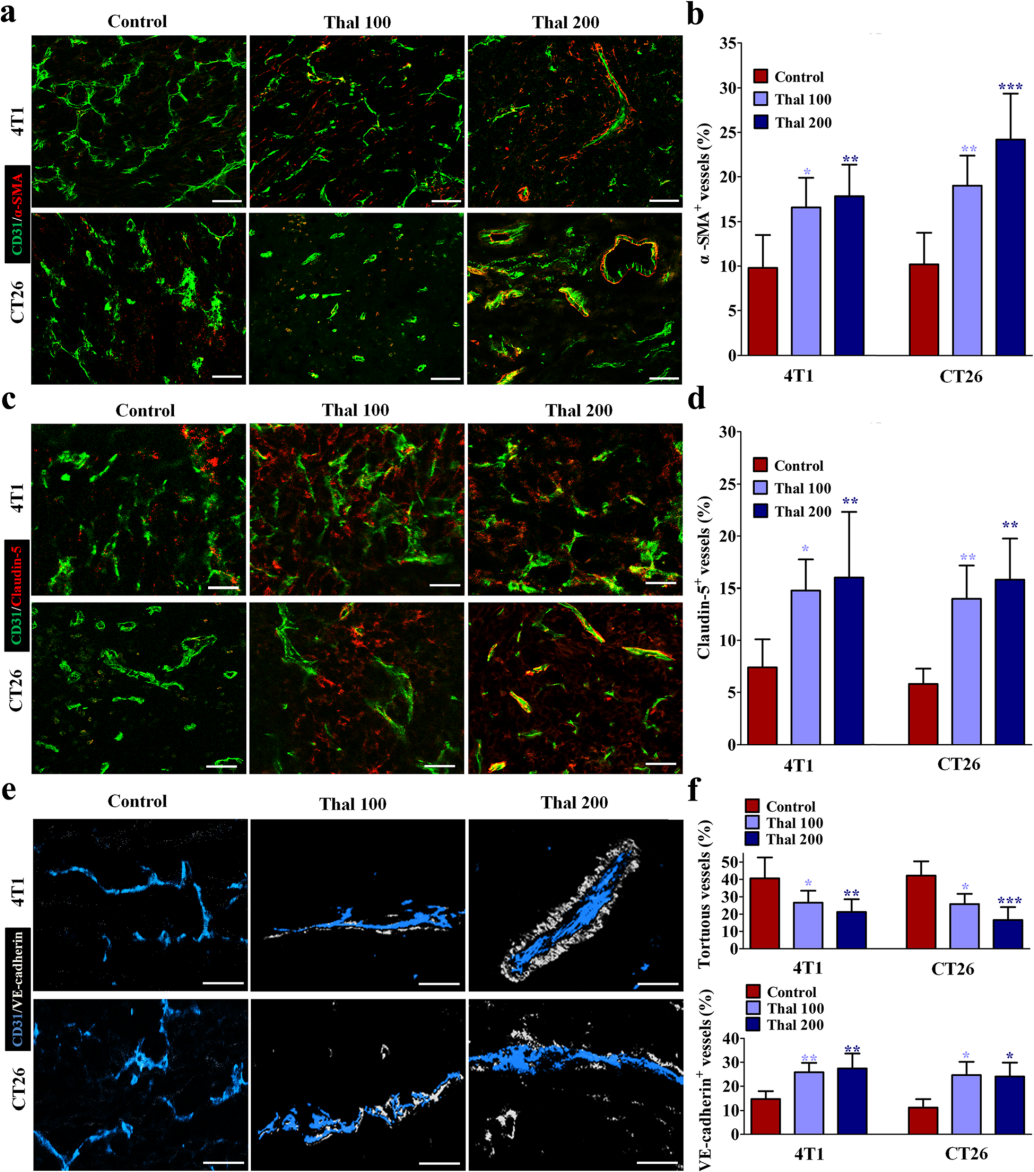
Thal increases pericyte coverage and endothelial tight-junctions. **a** Double staining for CD31 (Green) and α-SMA (Red) and quantification of percentage of α-SMA^+^ vessels in tumor sections from 4 T1 and CT26 models. Bars: 50 μm. **b** Staining for Claudin-5 (Red) indicating endothelial tight-junctions in tumor sections from 4 T1 and CT26 models. Tight-junctions are presented as a percentage of Claudin-5^+^/CD31^+^ vessels. Bars: 50 μm. **c** 4 T1 and CT26 tumors were stained for CD31 (Blue) and VE-cadherin (White) and quantified by percentage of tortuous vessels, and VE-cadherin^+^ vessels. Bars: 25 μm. Quantitative data are represented as mean ± SD. All *p* values were calculated by one-way ANOVA analysis followed by Dunnett’s multiple comparison test. *p < 0.05, ** *p* < 0.01, ***p < 0.001

In healthy microvessels, stationary pericytes stay and cover endothelial cells, but in tumor vessels, pericytes are activated and proliferate more actively, yet become separated from tumor endothelial cells, partly because of reduced adhesive properties and increased motility [34]. To determine how tumor vessel normalization occurred in animal models, we identified changes in tumor vessels, pericytes, tight-junctional molecules by immunostaining for the markers α-SMA, Claudin-5 and VE-cadherin, respectively (Fig. 3a-f) [35, 36]. In our analyses of structural changes of blood vessels in the tumor core region, we found that Thal dose-dependently increased α-SMA^+^ pericyte coverage, distribution of endothelial junctional molecules Claudin-5 and VE-cadherin on tumor vessels compared with control. In addition, the micro-vessels in Thal-treated tumors appeared less tortuous and more organized than in the control group. Together, these data sugessted that Thal stabilizes vessel structures by promoting the transformation of tumor vessels from abnormality to maturation in parallel with inhibition of tumor vessel tortuosity and thinning.

### Thal alters the vascular functionality

**Fig. 4:**
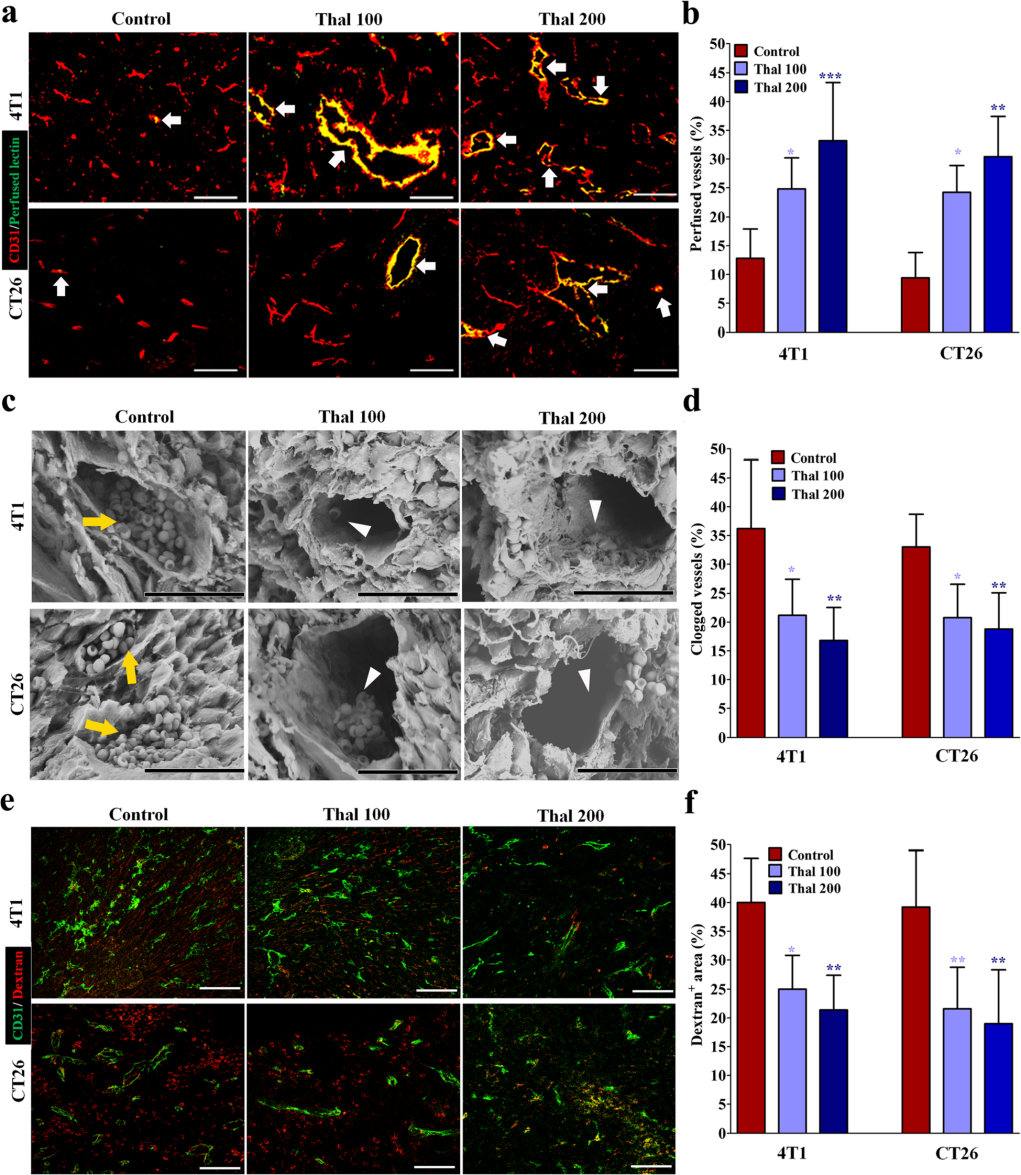
Thal enhances blood perfusion and supresses vascular destabilization. **a** Micrographs of FITC-conjugated lectin (Green) and CD31 (Red) stained vessels in tumor tissues from 4 T1 and CT26 models. FITC-lectin was intravenously injected at 30 min before euthanasia (*n* = 5). Arrowheads denote perfused vessels. Bars: 50 μm. **b** Quantification of vessel perfusion in 4 T1 and CT26 tumors. Lectin^+^ area is presented as a percentage per total sectional area. **c** Scanning electron microscopy and comparisons of the vascular congestion in 4 T1 and CT26 tumors. Yellow arrows indicate vessels plugged with red blood corpuscles; white arrowheads indicate open vascular lumens with less erythrocyte clot. Bars: 30 μm. **d** Quantification of percentage of clogged vessels in 4 T1 and CT26 tumors. **e** Images and comparison of dextran leakage of tumor vessels in 4 T1 and CT26 models. TRITC-dextran was intravenously injected at 30 min before euthanasia (*n* = 5). Bars: 50 μm. **f** Quantification of vessel leakage in 4 T1 and CT26 tumors. Dextran^+^ area is presented as a percentage per total sectional area. Quantitative data are represented as mean ± SD. All *p* values were calculated by one-way ANOVA analysis followed by Dunnett’s multiple comparison test. *p < 0.05, ** *p* < 0.01, ***p < 0.001

The improvement of vascular functionality is attributed to the normalization of vascular structure [37]. To examine the effect of Thal on vascular perfusion associated with tumor blood flow in vivo, lectin-FITC to label perfused vessels was injected into tumor-bearing mice and then stained 4 T1 and CT26 tumor sections for CD31. Thal dose-dependently increased the fraction of perfused lectin-FITC^+^ CD31^+^ vessels compared with the corresponding control group (Fig. 4a and b). Scanning electron microscopy was used to further investigate the effect of Thal on maintaining vascular perfusion. Tumors from control animals exhibited abnormal vessels, which were completely or partially plugged with red blood corpuscles and platelets. In contrast, vessels in the Thal-treated groups showed open lumens with less erythrocyte clot (Fig. 4c and d), suggesting that Thal alters the perfusion of tumor blood vessels. To analyze vessel functionality, we injected tumor-bearing mice with TRITC-conjugated dextran to measure leaky vessels and then stained 4 T1 and CT26 tumor sections for CD31 to identify all vessels so that the percentage of leaky vessels could be determined. Vessels in the Thal-treated groups showed significantly decreased dextran leakage as compared with control group (Fig. 4e and f), indicating that Thal treatment make the endothelial cell barrier more impenetrable. These results collectively indicated that Thal enhances vessel functionality by increasing the tumor perfusion and decreasing the vascular leakiness, thereby contributing to normalization of tumor vessels.

### Thal enhances the delivery and efficacy of chemotherapy

**Fig. 5:**
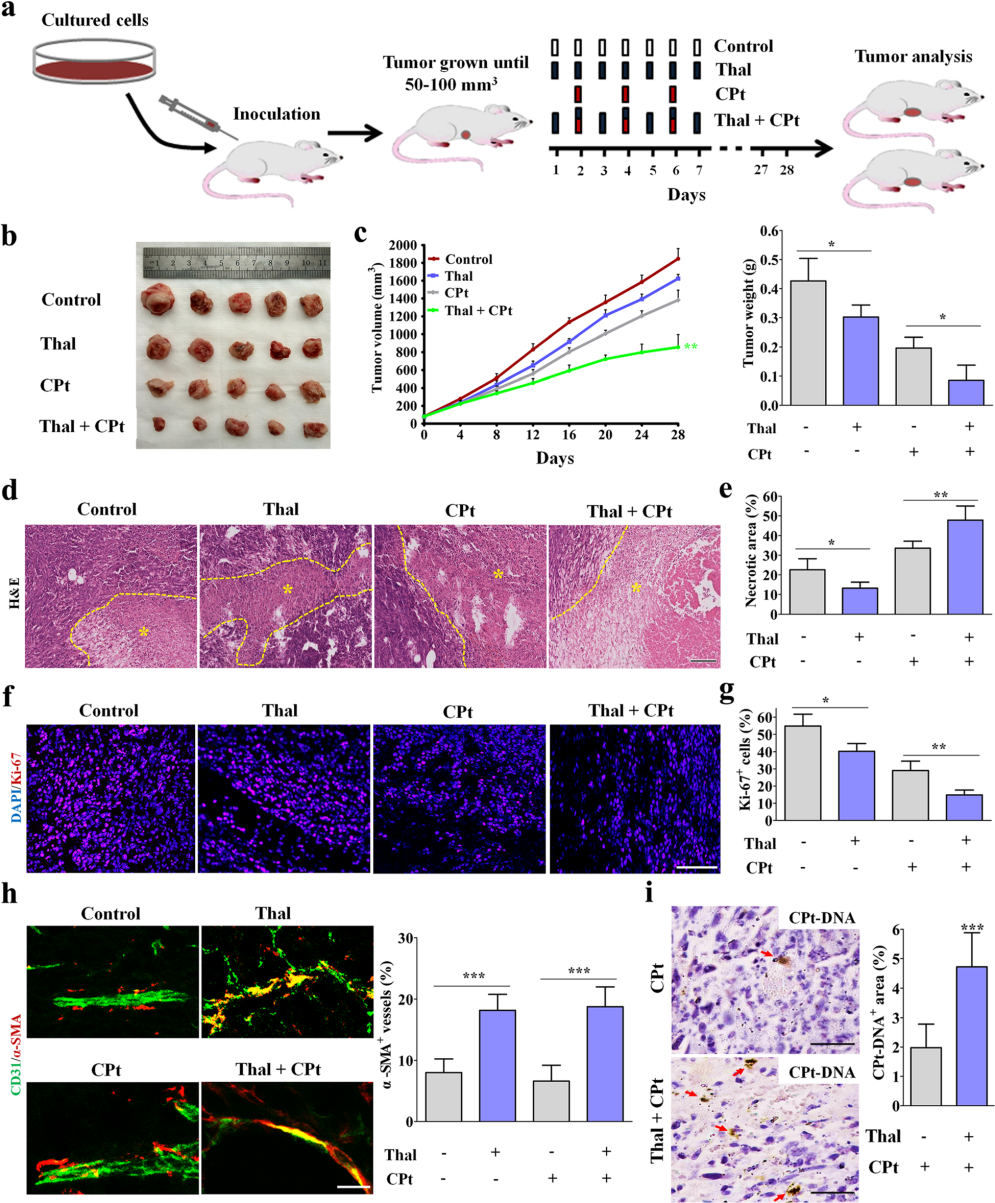
Thal increases the delivery and efficacy of chemotherapy by tumor vessel normalization. **a** Schematic diagram depicting generation of 4 T1 tumor homograft model and schedules for injection and analyses. When tumors were approximately 50–100 mm^3^, tumor-bearing mice were intraperitoneally injected with vehicle, Thal, CPt or Thal + CPt at indicated time points. **b** Visualized tumors in 4 T1 tumor model after resection. Five tumor tissues per group were showed. **c** Quantification of tumor volume and tumor weight. **d** H&E-stained images of tumor sections and comparison of necrotic areas (Yellow dotted line and asterisk) in 4 T1 tumors harvested on day 28. Bar: 200 μm. **e** Quantification of necrotic areas in 4 T1 tumors. Necrosis is presented as a percentage per total sectional area. **f** Micrographs of sections from 4 T1 tumors stained for proliferation marker Ki67 (Red). Nuclei are counterstained with DAPI. Bar: 100 μm. **g** Quantification of percentage of Ki67^+^ cells. **h** Micrographs of 4 T1 tumor sections from control, Thal, CPt and Thal + CPt-treated mice, stained for CD31 and α-SMA to assess pericyte coverage. Bar: 25 μm. **i** Micrographs and comparison of CPt^+^ area in 4 T1 tumors harvested on day 28. CPt-DNA adducts were immunostained (Arrows), and CPt^+^ area is measured as a percentage per total sectional area. Bars: 50 μm. Quantitative data are represented as mean ± SD. Student’s t test was utilized for comparision of two groups, one-way ANOVA analysis followed by Bonferroni test was used for multiple comparisons. *p < 0.05, ** *p* < 0.01, *** *p* < 0.001

The effectiveness of chemotherapy depends largely on the dose that can be available in situ [38]. Previous studies have shown that tumor vessel normalization enhances the delivery and efficacy of chemotherapy [39]. Based on the profound effect of Thal on reversing the dysfunctional and disorganized tumor vessels, we therefore explored if treatment of 4 T1 tumor-bearing mice with Thal enhanced the efficacy of a suboptimal CPt dose (2.5 mg/kg every other day), which affected tumor growth only minimally (Fig. 5a) [40]. When tumors were approximately 50–100 mm^3^, mice were injected with 200 mg/kg/d of Thal alone or together with CPt (2.5 mg/kg, q.a.d); control mice were injected with vehicle (Fig. 5a). Consistent with our expectation, the combination of Thal with this dose of CPt caused a greater suppression of tumor growth compared with either drug alone (Fig. 5b and c). The synergistic anti-tumor effect of Thal combined with CPt was confirmed by tumoral tissue sections, which showed that the combination of Thal and CPt signifcantly increased necrosis (Fig. 5d and e) and reduced the tumor Ki-67 proliferative index (Fig. 5f and g) comparing CPt monotherapy group. To investigate the effects of Thal and CPt on tumor vessel normalization, we stained for markers of tumor vessel and pericytes. Compared with CPt alone, the combination of Thal and CPt increased pericyte coverage (Fig. 5h). We also determined the intra-tumoral diffusion of CPt by monitoring assessment of CPt-DNA-adducts formation. The CPt-DNA-adducts positive area was significantly greater in the the combination of Thal and CPt group compared with CPt monotherapy group (Fig. 5i), suggesting the improved chemotherapeutic effect was due to more efficient delivery of CPt. Collectively, these resluts indicated that Thal enhances the delivery of CPt through tumor vessel normalization, an observationin accordance with the increased tumor perfusion.

### Combined treatment of Thal and CPt delays metastasis and prolongs overall survival

**Fig. 6:**
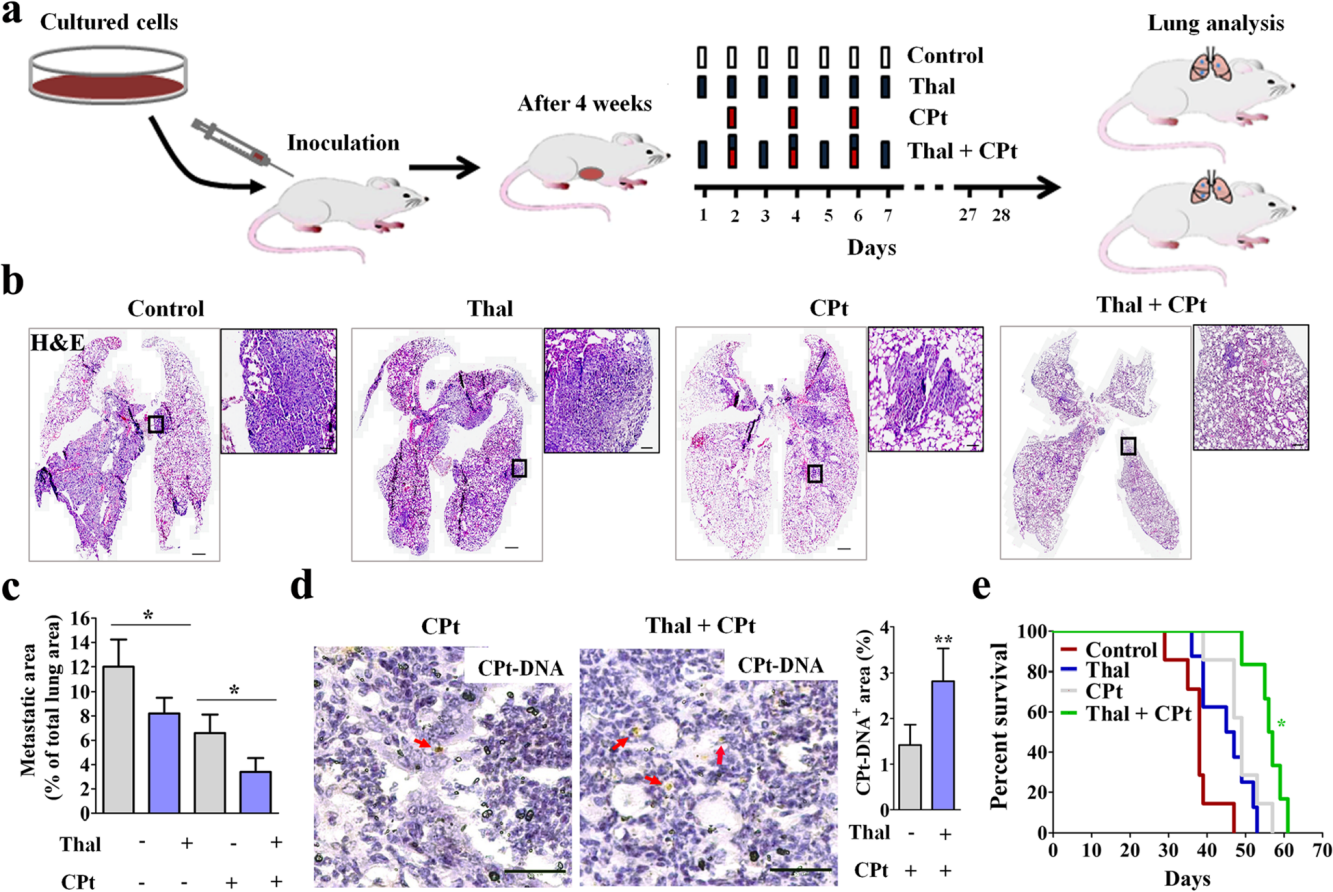
Thal combined with CPt suppresses metastasis and prolongs overall survival. **a** Schematic diagram depicting generation of 4 T1 spontaneous metastasis model and schedules for injection and analyses. Four weeks after 4 T1 cells inoculation, tumor-bearing mice were given intraperitoneal injections of vehicle, Thal, CPt or Thal + CPt at indicated time points. **b** Micrographs of lung sections stained for H&E. Metastatic colonies are viewed under higher magnification (Right panels). Bars: 500 μm (Left), 50 μm (Right). **c** Quantification of metastatic nodule area presented as a percentage of total lung section area. **d** Micrographs and comparison of CPt^+^ area in lungs. CPt-DNA adducts were immunostained (Arrows), and CPt^+^ area is measured as a percentage per total sectional area. Bars: 50 μm. **e** Kaplan-Meier survival curves of mice on 4 T1 spontaneous metastasis model (*n* = 8). Data were analyzed by the log-rank test. *p < 0.05

To establish a spontaneous metastasis model, 4 T1 cells were injected into the fourth mammary fat pad of Balb/c mice [41]. After 4 weeks post-injection, vehicle, Thal (200 mg/kg/d), CPt (2.5 mg/kg, q.a.d) and Thal plus CPt were injected intraperitoneally (Fig. 6a). H&E staining showed 4 T1 tumor cell invasion and colonization in lung tissues of vehicle, Thal-treated and CPt-treated mice, but slight tumor cell invasion and colonization in combination of Thal and CPt group after 28 days (Fig. 6b). Of note, combination treatment showed the greatest attenuation of metastasis compared with both mono-treatment groups (Fig. 6c). Intriguingly, we observed more CPt-DNA-adducts in pulmonary metastasis focuses of mice treated with combination of Thal and CPt compared to CPt alone (Fig. 6d), indicating that CPt delivery was increased. The Kaplan-Meier survival assay showed that the cumulative survival rate and median survival time in mice treated with combination of Thal and CPt were significantly increased compared with that in mice treated with drug alone (log-rank tests) (Fig. 6e). Thus, Thal enhances the anti-tumor effect of CPt and prolongs tumor-bearing mice survival time.

### Thal modulates angiogenesis-related cytokines

**Fig. 7:**
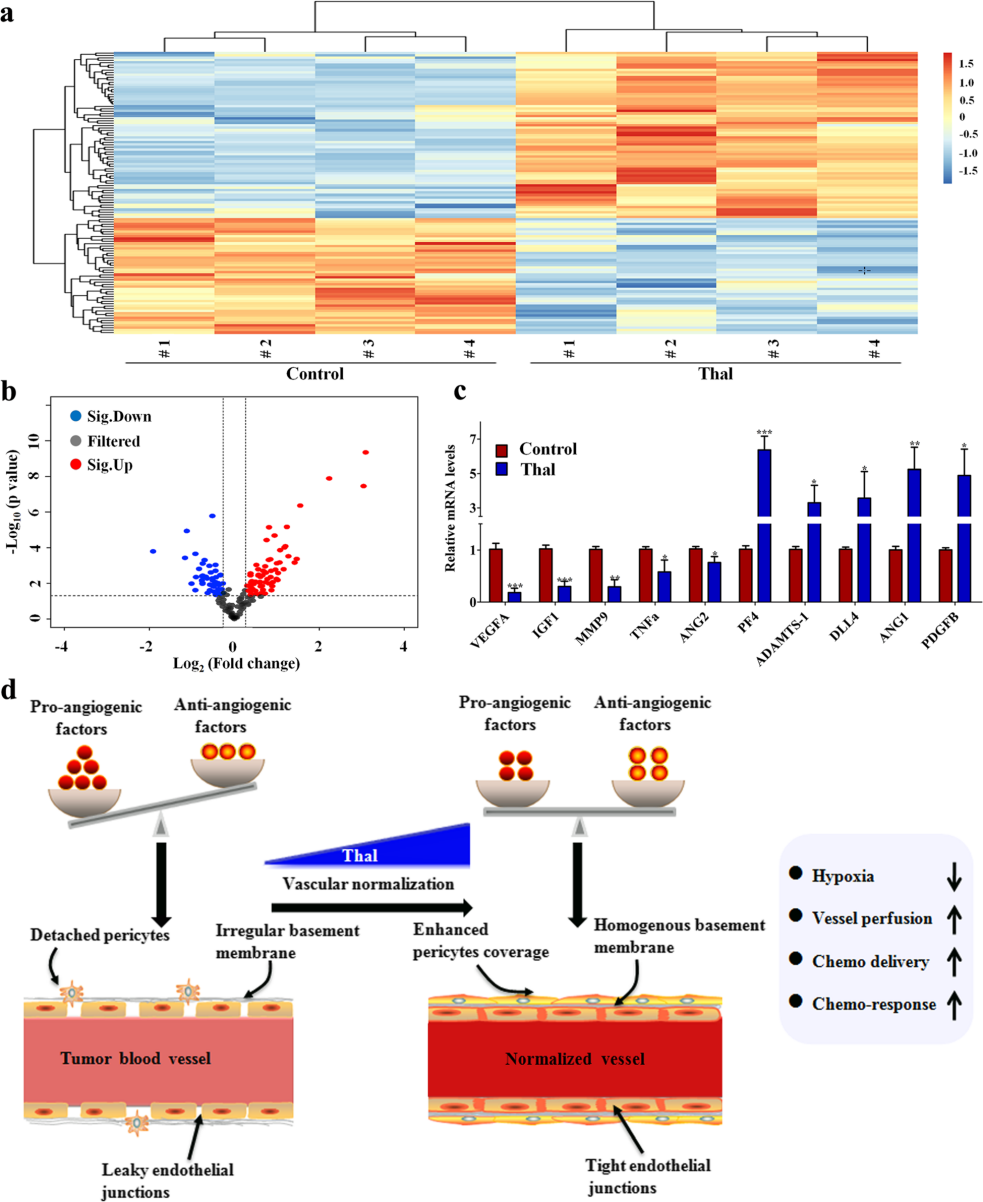
Thal regulates vascular stabilization signature proteins. **a** Tumor lysates from control and Thal-treated group were applied to the angiogenesis antibody array (*n* = 4). A heatmap was used to classify the protein expression patterns in Thal-treated tumors compared to control tumors. Red color indicates upregulation and blue indicates downregulation. **b** Volcano plot of differentially expressed proteins between the Thal and control groups. The *y*-axis indicates the negative logarithm of the *p*-value; the *x*-axis indicates the base 2 logarithm of fold change. **c** The transcriptional levels of indicated angiogenesis-related factors in harvested tumors were measured by qRT-PCR (*n* = 3). β-actin was used as the control. Values are mean ± SD. *p < 0.05, ** *p* < 0.01, ***p < 0.001. **d** Schematic diagram depicting changes of tumor vascular system by Thal-induced vascular normalization in solid tumor. During tumor development, the tumor vascular abnormalities stem from an imbalance between angiogenic growth factors and angiogenic inhibitors in the local milieu. The abnormal tumor vessels are characterized by a disorganized and tortuous architecture with highly dysfunctional pericytes and leaky endothelial junctions, which impair tumor perfusion, and render it more hypoxic and chemoresistance (Left). By decreasing diverse pro-angiogenic factors and inducting vascular stabilization signature factors, Thal corrects the imbalance between pro- and anti-angiogenic factors and transforms abnormal tumor vasculatures towards a more inerratic and functional state. These phenotypic changes lead to decreased leakage, enhanced perfusion, and reduced hypoxia. Eventually, delivery of chemotherapeutic drugs was highly enhanced through the Thal-induced vascular normalization, leading to profound anti-tumor effects (Right)

Excessive pro-angiogenic factors are a powerful driving force for abnormal angiogenesis, leading to an immature vascular system [13, 42]. To uncover the mechanisms through which Thal was capable of normalizing the tumor vasculature, we analyzed the levels of expression of a series of angiogenic factors by an angiogenesis-related protein array on wholeprotein extracts of tumors. The 117 detected proteins are presented in Additional file 2: Table S1, and are also displayed in heat map format for ease of comparison (Fig. 7a). It is evident from these results that Thal-treated tumors and control tumors have distinct protein profiles. A total of 61 proteins were upregulated and 56 proteins were downregulated in Thal-treated tumor tissues compared with control (Fig. 7b). For example, Thal significantly reduced the expression of diverse pro-angiogenic factors including VEGFA, angiopoietin 2 (ANG2), matrix metallopeptidase 9 (MMP9), insulin-like growth factor 1 (IGF1) and tumor necrosis factor α (TNFα), while increasing the expression of several important anti-angiogenic factors including platelet factor 4 (PF4), delta-like ligand 4 (DLL4) and a disintegrin and metalloproteinase with thrombospondin motifs-1 (ADAMTS-1). Intrestingly, Thal notably increased a set of proteins known to regulate the processes of vascular maturation, such as ANG1 and platelet-derived growth factor (PDGFB). Real-time quantitative PCR was used to verify the initial screening results. The changes of angiogenesis-related genes between Thal-treated and control tumors detected by PCR were consistent with the results of antibody microarray (Fig. 7c). Notably, ANG1 is mainly secreted by endothelial cells, pericytes, and cancer cells, playing a key role in regulating vasculature [43]. ANG1 serves as a stabilizer for tumor vasculature by increasing the ratio of pericyte coverage [44]. PDGFB also is a key regulator of mural cell recruitment, acting as an attractant for migrating pericytes expressing PDGF receptor-β (PDGFR-β) [45, 46]. This raises the question of whether Thal affects the expression of signaling related to vascular stabilization in both endothelial cells and tumor cells. We found that Thal increased the levels of ANG1 and PDGF-B not only in tumor cells, but also in endothelial cells (Additional file 3: Figure S2). Taken together, these findings indicated that Thal may “reprogram” the angiogenesis-related protein expression pattern, shifting from a pro-angiogenic profile into a vascular stabilizing signature in tumors.

## Discussion

Early pioneers of angiogenic research observed over a century ago that growth of solid tumors beyond a few millimeters strongly requires new blood vessels [7]. Angiogenesis within solid tumors is driven largely by hypoxia, which is a hallmark of TME [13, 14, 47]. However, compared with the normal vascular system, hypoxia-triggered the abundance of VEGF and other pro-angiogenic factors in TME leads to continual angiogenesis and the production of an a structurally and functionally abnormal tumor vasculature [37]. Anatomically, these abnormal tumor vessels are characterized by an irregular, tortuous and disorganized architecture with a highly dysfunctional and leaky endothelial cell layer. Functionally, the tumor vasculature is poorly perfused due to destabilized vascular structure and increased IFP, leading directly to severe hypoxia and insufficient drug delivery. These physiological changes in turn provoke tumorigenesis, distant metastasis, and chemoresistance [7]. For these reasons, strategies aimed at restoring tumor vascular system to a more normal state are an importance issue that is currently under extensive exploration. Improved perfusion and chemotherapy delivery by anti-angiogenic therapy has proven effective in multiple mice models and subpopulations of diverse cancer patients [7, 48].

In recent years, the invention of Thal represents an important advancement in the anti-angiogenic strategies for anticancer therapies. We have previously reviewed the anti-angiogenic and anti-tumor effects of Thal on various neoplastic diseases [28]. Moreover, Thal and its analogs have been successfully used in treatment of immune disorders including graft-versus-host disease and autoimmune diseases [28, 49]. Recently, it has been reported that combined administration of Thal and chemotherapeutic drug exhibited synergistic effect in breast cancer models [29]. Thal has also been used combination with other chemotherapy drugs in treatment of multiple myeloma [50]. However, it is not yet known whether the synergistic effect of Thal combined with other cytotoxic drugs is attributable to tumor vascular normalization. Hence, the tumor vascular normalization effects of Thal demonstrated here help to explain the anti-tumor and chemo-sensitization effects of Thal in solid tumors.

Using two highly vascularized homograft models, we demonstrated that the anti-tumor effect of Thal is not reliant on direct tumoricidal activity but relies on an effect on tumor vessels, raising the question of the specific anti-tumor mechanism. We then investigated the efficacy of Thal on tumor vascular normalization, specifically including physiological characteristic, morphological structure and maturation of tumor vessels.

Hypoxia provides a niche for cancer stem cells and promotes inflammation, while also engendering resistance to many widely used therapeutic drugs [7]. Thus, alleviating hypoxia by tumor vascular normalization is critical for improving cancer treatment. Our study demonstrated that Thal resulted in a marked reduction of intratumoral hypoxia. Thal also significantly inhibited tumor vascular permeability. Thus, Thal could be a promising therapeutic option that generates a favorable TME.

It has been reported that direct restoration of endothelial junctions and preservation of the vascular integrity could be a significant strategy to induce normalization of tumor vessels and improve tumor perfusion [51]. In this study, we demonstrated that Thal not only inhibits vascular instability, but also restores structural and functional vascular intergrity, resulting in normalization of tumor vessels. Indeed, Thal not only promotes pericyte coverage but also tightens endothelial junctions of vessels at the tumor core region. The normalized tumor vasculature leads to reduced MVD, decreased vessel tortuosity, increased vessel diameter and thickness. They also decreased vessel leakage and enhanced perfusion, which culminates in favorable changes in the whole TME to hinder tumor progression and dissemination. Eventually, the delivery of chemotherapeutic drug was highly enhanced through the normalized tumor vasculature, thus resulting in profound anti-tumor and anti-metastatic effects (Fig. 7d). These morphological and functional parameters are consistent with the concept of vessel normalization, as initially proposed as a goal to improve blood supply and delivery of chemotherapeutic agents by anti-angiogenic drugs [52]. In this regard, our findings provide direct evidence of the tumor vascular normalization effect of Thal and may thus help to establish a schedule for combination therapy in the treatment of patients with solid tumors.

In normal tissue, the effects of pro-angiogenic factors, like VEGF, IL-8 and bFGF, are exquisitely counterbalanced by that of endogenous anti-angiogenic molecules, such as VEGFR1 and thrombospondins. During tumor development, the balance is tipped in favor of pro-angiogenic molecules, resulting in a structurally and functionally abnormal vasculature network [37]. Thus, tip the imbalance between pro- and anti-angiogenic factors back toward equilibrium could normalize tumor vasculature. In present study, we measured angiogenic proteins in tumor lysate indicating a regulatory effect of Thal on the expression of various angiogenic factors. Among these factors, expressions of VEGFA, ANG2, MMP9, IGF1 and TNFα, which are very important for almost all steps in the angiogenesis process, were significantly reduced in tumors treated with Thal. In contrast, several anti-angiogenic factors, including PF4, DLL4 and ADAMTS-1, were upregulated by Thal in tumors. In particular, Thal increased the expressions of ANG1 and PDGF-B in both tumor cells and endothelial cells. ANG1 is generally considered a Tie-2 agonist which promotes vessel maturity and stability and reduces leakiness [53]. PDGFB is a well characterized recruitment signal for pericytes, expressing PDGFR-β. Ablation of PDGFB or its receptor, or removal of its retention signal leads to abnormal vessel leakage [54]. From this point of view, our findings indicated that Thal may “reprogram” the angiogenesis-related protein expression pattern, shifting from a pro-angiogenic profile into a vascular stabilizing signature in tumors. Collectively, Thal corrects the imbalance between pro- and anti-angiogenic factors and converts abnormal tumor vascular properties into a more inerratic and functional state.

Thal has an unenviable history of disrepute owing to its teratogenic effects. However, of late, the efficacy in suppressing angiogenesis may have earned Thal and its analogues a place as potential therapeutic agents [55]. The current study has demonstrated that Thal combined with cytotoxic drug exerts a profound anti-tumor effect, which provides a new promising therapeutic strategy for solid tumors. Importantly, it has been investigated for clinical effects of Thal in combination with other chemotherapeutic agents or targeted therapeutic agents in the treatment of patients with solid tumors, such as lung cancer, prostate cancer and primary hepatocellular carcinoma [56–58]. By providing these experimental results, we suggest that Thal improves delivery and efficacy of chemotherapeutic agents in solid tumors through tumor vascular normalization. Further clinical and pre-clinical studies remain to be done to demonstrate its safety and efficacy.

## Conclusions

In summary, our findings provide direct evidence that Thal remodels the abnormal tumor vessel system into a normalized vasculature by restricting excessive vessel sprouting, increasing pericyte coverage, tightening endothelial junctions and enhancing tumor perfusion, eventually resulting in an increased delivery and efficacy of chemotherapeutic drug. Our results may lay solid foundation for the development of Thal as a novel candidate agent to maximize the therapeutic efficacy of conventional chemotherapeutic drugs for solid tumors. However, optimal designs of drug scheduling and effective imaging techniques are absolutely indispensable to achieve maximal outcome.

## Additional files

Additional file 1: **Figure S1.** Effect of Thal on tumor cell proliferation. (a) The tolerance of 4 T1 and CT26 cells to Thal was determined by MTT assay. (b) Clonogenic growth of 4 T1 and CT26 cells in the absence or presence of Thal. Clones were stained with crystal violet (left). Numbers of clones are presented as mean ± SD of three independent experiments (right). (TIF 2354 kb)

Additional file 2: **Table S1.** List of differentially expressed proteins between control tumors and Thal-treated tumors. (XLS 46 kb)

Additional file 3: **Figure S2.** Thal upregulates ANG1 and PDGFB in both tumor cells (a) and endothelial cells (b). All data are presented as mean ± SD of triplicates. *p < 0.05, ** *p* < 0.01. (TIF 762 kb)

## Data Availability

The datasets used and analysed during the current study are available from the corresponding author on reasonable request.

## Abbreviations

ADAMTS-1: A disintegrin and metalloproteinase with thrombospondin motifs-1
ANG: Angiopoietin
ATCC: American Type Culture Collection
bFGF: Basic fibroblast growth factor
CPt: Cisplatin
DLL4: Delta-like ligand 4
DMSO: Dimethyl sulfoxide
FBS: Fetal bovine serum
FDA: Food and Drug Administration
HUVEC: Human umbilical vein endothelial cell
IFP: Interstitial fluid pressure
IGF1: Insulin-like growth factor 1
MMP9: Matrix metallopeptidase 9
MVD: Micro-vessel density
PCR: Polymerase chain reaction
PDGFB: Platelet-derived growth factor
PF4: Platelet factor 4
SD: Standard deviation
Thal: Thalidomide
TME: Tumor microenvironment
TNFα: Tumor necrosis factor α
VEGF: Vascular endothelial growth factor
