# Supplementary figures and images for: Tumor vasculature remolding by thalidomide increases delivery and efficacy of cisplatin
Source: J Exp Clin Cancer Res. 2019 Oct 28;38:427. doi: 10.1186/s13046-019-1366-x (PMC6816178; doi:10.1186/s13046-019-1366-x)

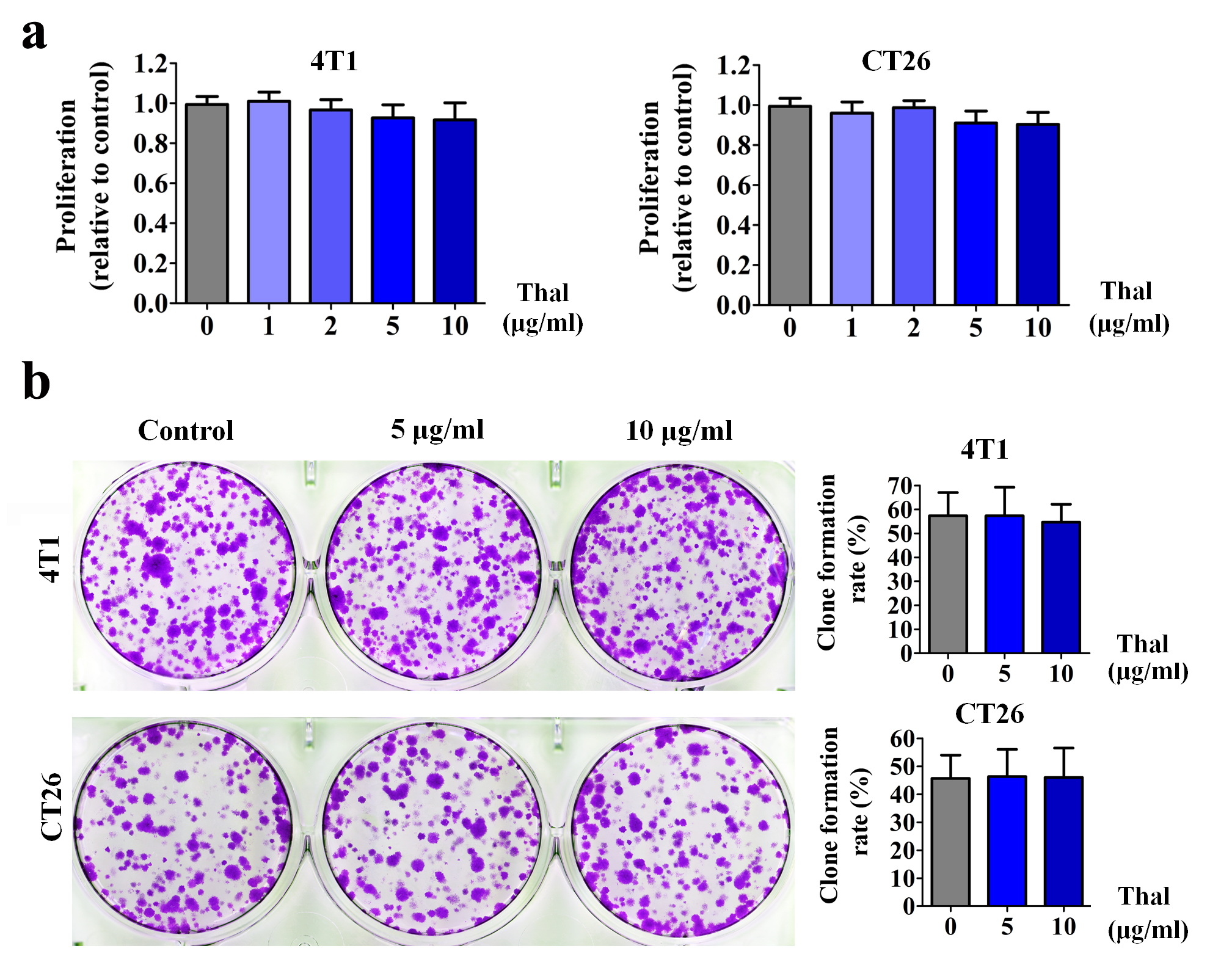

Supplement: Supplementary file 1 — Figure S1. Effect of Thal on tumor cell proliferation. (a) The tolerance of 4 T1 and CT26 cells to Thal was determined by MTT assay. (b) Clonogenic growth of 4 T1 and CT26 cells in the absence or presence of Thal. Clones were stained with crystal violet (left). Numbers of clones are presented as mean ± SD of three independent experiments (right). (TIF 2354 kb) [file 13046_2019_1366_MOESM1_ESM.tif]

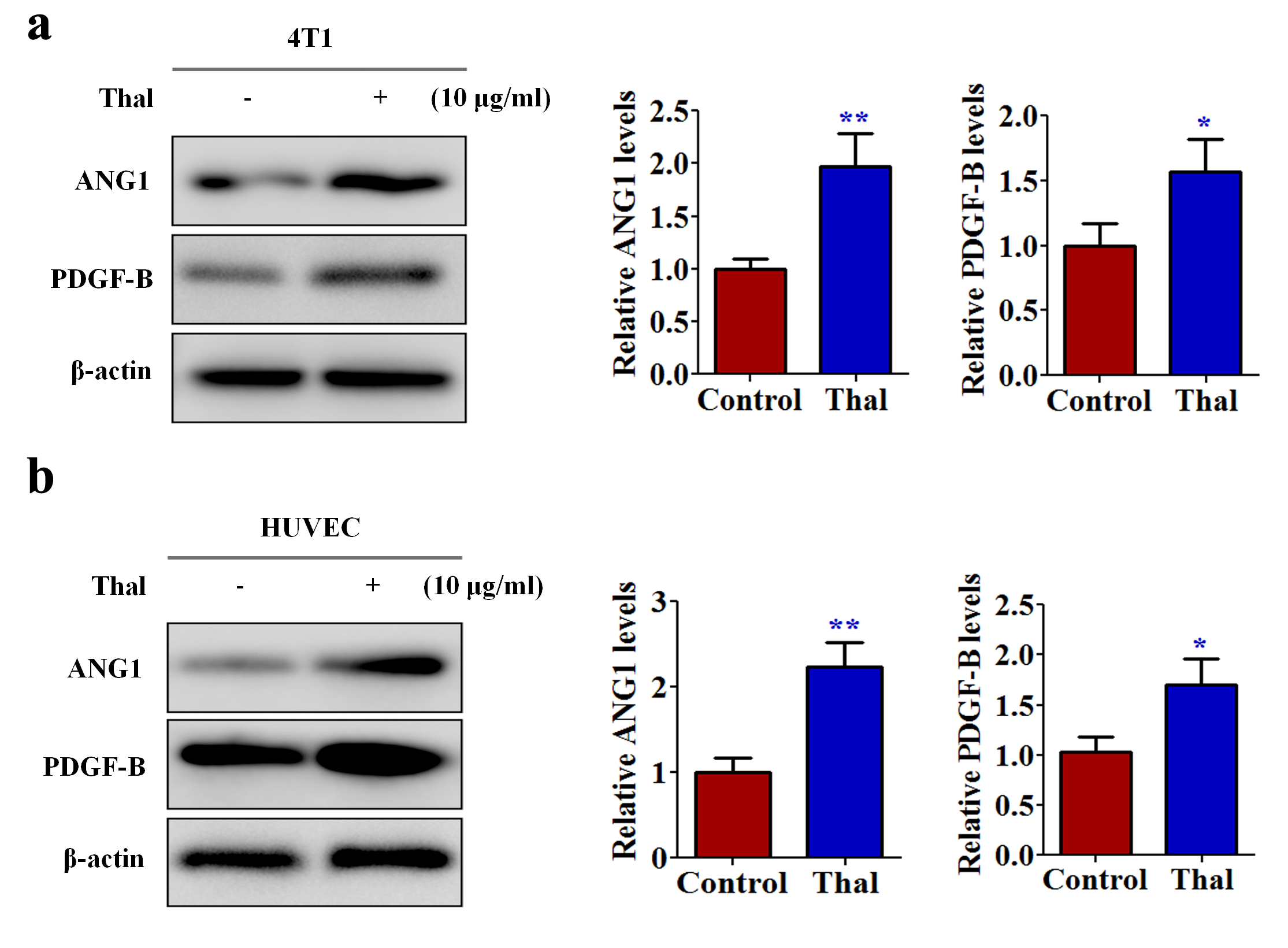

Supplement: Supplementary file 3 — Figure S2. Thal upregulates ANG1 and PDGFB in both tumor cells (a) and endothelial cells (b). All data are presented as mean ± SD of triplicates. *p < 0.05, ** p < 0.01. (TIF 762 kb) [file 13046_2019_1366_MOESM3_ESM.tif]
